# Intravenous Contrast‐Enhanced MR Myelography in CSF Leakage for the Detection of Spinal CSF Lamellae

**DOI:** 10.1111/jon.70056

**Published:** 2025-05-27

**Authors:** Zeynep Bendella, Robert Haase, Ralf Clauberg, Stefan Zülow, Christine Kindler, Alexander Radbruch, Daniel Paech, Katerina Deike

**Affiliations:** ^1^ Department of Neuroradiology, University Medical Center Bonn Rheinische Friedrich‐Wilhelms‐Universität Bonn Bonn Germany; ^2^ Clinical Neuroimaging Group German Center for Neurodegenerative Diseases (DZNE) Bonn Germany; ^3^ Section of Vascular Neurology, Department for Neurology Universitätsklinikum Bonn Bonn Germany; ^4^ Department of Old Age Psychiatry and Cognitive Disorders, University Hospital Bonn University of Bonn Bonn Germany; ^5^ Department of Radiology Brigham and Women's Hospital, Harvard Medical School Boston Massachusetts USA; ^6^ Athinoula A. Martinos Center for Biomedical Imaging Massachusetts General Hospital Charlestown Massachusetts USA

**Keywords:** CSF leakage, epidural lamella, heavily T2‐weighted (HT2) FLAIR imaging, intracranial hypotension, spinal CSF lamella

## Abstract

**Background and Purpose:**

Intracranial hypotension (IH) results from cerebrospinal fluid (CSF) leakage from the dural sac, occurring spontaneously or iatrogenically (e.g., post‐lumbar puncture), and may cause a wide range of symptoms with significant functional impairment. Accurate detection of the epidural CSF lamella is key to diagnosis. This study evaluated the diagnostic value of intravenous contrast‐enhanced MRI using heavily T2‐weighted FLAIR (HT2‐FLAIR) spine imaging compared to nonenhanced MR myelography at 3 Tesla.

**Methods:**

Ten consecutive patients with IH symptoms were prospectively examined using HT2‐FLAIR imaging of the spine before and up to 3 h after gadolinium‐based contrast agent administration, alongside noncontrast MR myelography. Two readers assessed the conspicuity of the CSF lamella on contrast‐enhanced HT2‐FLAIR (ceHT2‐FLAIR) using a score from −2 to +2 and evaluated additional diagnostic benefit.

**Results:**

A CSF lamella was seen in eight of 10 patients as a strongly enhancing band on ceHT2‐FLAIR. In one case, the lamella was visible exclusively on ceHT2‐FLAIR (conspicuity score [CS] = 2, *n* = 1) and was more conspicuous in three cases (CS = 1, *n* = 3). Six cases showed equal conspicuity (CS = 0, *n* = 6). In two cases each, ceHT2‐FLAIR either enabled diagnosis or provided supporting information. In six cases, it confirmed diagnosis based on noncontrast imaging. Beyond improved conspicuity, ceHT2‐FLAIR helped detect low‐flow leaks, optimize axial slice positioning, and assess CSF lamella distribution.

**Conclusions:**

Intravenous ceHT2‐FLAIR MRI may be considered as an additional tool in CSF leak evaluation, particularly when used for detecting indirect signs of IH.

## 1 Introduction

Intracranial hypotension (IH) can occur spontaneously or iatrogenically, for example, following a lumbar puncture. Both forms share the same clinical symptoms, which can vary depending on the location and severity of the leak. A hallmark symptom is a persistent, positional headache that worsens in an upright position and improves when lying down. Other common symptoms include nausea, vomiting, and auditory changes such as tinnitus or hearing loss, and some patients report disturbances in consciousness, concentration difficulties, and an increased heart rate. Visual changes, including blurred vision, double vision, or light sensitivity, may occur if the leak affects the optic nerve [1, 2, 3].

As cerebrospinal fluid (CSF) leaks can lead to significant impairment of daily functioning, nontargeted epidural blood patching is performed when a CSF lamella is detected, particularly in patients who do not experience lasting symptom relief from purely conservative measures [4, 5, 6, 7, 8].

Since clinical presentation can vary, imaging plays a crucial role in establishing the diagnosis. Currently available imaging tools for detecting CSF leaks include CT myelography, digital subtraction myelography, radioisotope cisternography, and noncontrast and intrathecal gadolinium‐enhanced MR myelography. While CT myelography is widely recognized by radiologists for IH workup due to its high spatial resolution, ability to depict degenerative changes, and capability to measure CSF opening pressure, its disadvantages include radiation exposure and the need for a lumbar puncture [9, 10, 11, 12, 13, 14, 15].

Spinal MRI can aid in diagnosing IH by depicting epidural and paraspinal CSF accumulations. However, detecting CSF lamellae on noncontrast MR myelography can pose significant challenges due to their often subtle and elusive nature, as well as spinal CSF flow artifacts and the artificial suppression of epidural fat. MR myelography following intrathecal gadolinium‐based contrast agent (GBCA) is an off‐label use [12], and the lumbar puncture may aggravate patients’ symptoms [16, 17].

To facilitate the detection of CSF lamellae, we evaluated an intravenous contrast‐enhanced MRI sequence that does not require a lumbar puncture and does not involve off‐label use. Heavily T2‐weighted FLAIR MRI (HT2‐FLAIR) has gained attention for its high sensitivity to even low concentrations of GBCAs [18]. Fluid‐attenuated inversion recovery (FLAIR) suppresses the signal from unenhanced CSF, thereby enhancing the visibility of hyperintense structures against a dark background. Additionally, it offers greater sensitivity than T1‐weighted imaging for detecting subtle T1 shortening caused by contrast agents, highlighting regions with contrast leakage. These properties make this technique a promising tool for detecting CSF leaks, particularly since the underlying pathophysiology involves disruption of the highly vascularized dura.

Therefore, the aim of the present study was to further assess the clinical value of intravenous contrast‐enhanced MR myelography with acquisition of a GBCA‐enhanced HT2‐FLAIR sequence (ceHT2‐FLAIR).

## 2 Methods

### 2.1 Patients

Between October 2020 and October 2023, 10 consecutive patients (mean age ± standard deviation, 42.83 ± 13.84 years, nine women) who were admitted to the University of Bonn Medical Center and were suspicious of IH according to the criteria proposed by Shievink [19] or the Headache Classification Committee of the International Headache Society, 3rd edition [20] were prospectively included in the study if the contrast‐enhanced MRI was clinically indicated and written informed consent was obtained. The study design and protocol were approved by the Ethics Committee of the University of Bonn. Patient characteristics are summarized in Table 1.

**TABLE 1:** Patient demographics and characteristics.

Patient number	Age (years)	Symptoms	Presence of epidural lamella on noncontrast MR myelography	Extension of epidural lamella on noncontrast MR myelography	Type of intracranial hypotension
1	18	Orthostatic headache, pain and stiffness in the neck	None	None	Iatrogenic after lumbar puncture
2	42	Orthostatic headache, sensitivity to light	Yes	Cervical and thoracic spine	Spontaneous
3	39	Orthostatic headache, sensitivity to light	Yes	Cervicothoracic junction	Spontaneous
4	32	Orthostatic headache, pain and stiffness in the neck	Yes	Thoracic spine	Spontaneous
5	37	Orthostatic headache, nausea	Yes	Thoracic spine	Iatrogenic after lumbar puncture
6	46	Orthostatic headache, pain and stiffness in the neck	Yes	Cervical up to lumbar spine	Spontaneous
7	39	Orthostatic headache, nausea	Uncertain	Lower cervical spine to lower thoracic spine	Spontaneous
8	48	Orthostatic headache, sensitivity to light	None	None	Iatrogenic after lumbar puncture
9	44	Orthostatic headache, nausea	None	None	Iatrogenic after lumbar puncture
10	57	Orthostatic headache, visual impairments	Yes	Cervical up to lumbar spine	Iatrogenic after lumbar puncture

### 2.2 MRI

MRI was performed on a clinical whole‐body 3 Tesla MRI system (Achieva TX, Philips Healthcare, Best, The Netherlands) equipped with a phased‐array spinal coil. All patients with IH underwent MRI of the brain and the whole spine. All examinations of the spine consisted of the following sequences: sagittal T2 modified DIXON (mDIXON), sagittal T1, and pre‐ and postcontrast sagittal HT2‐FLAIR images. Scanning parameters are summarized in Table 2.

**TABLE 2:** Sequence parameters of spinal MRI.

Sequence	HT2‐FLAIR	T2w mDIXON	T1w
Pulse type	3D turbo spin echo	Turbo spin echo	Spin echo
Orientation	Sagittal	Sagittal	Sagittal
TR (ms)	4800	2069	838
TE (ms)	500	110	15
Voxel size (mm)	1.1 × 1.1 × 1.6	0.9 × 1.05 × 3	0.8 × 1 × 4
Matrix (mm)	252 × 181	200 × 261	224 × 276
Inversion time (ms)	1550	—	—
Flip angle	90	90	80
Echo train length	255	25	7
Sagittal slices	87	15	15
Slice thickness (mm)	1.6	3	4
In‐plane resolution (mm)	1.2	0.9	0.8
Bandwidth (Hz/pixel)	992.1/0.438	401.4/1.082	307.6/1.412
Acceleration factor	35	17	64
Number of excitations	2	1	1
Total time (min)	5:41	2:25	1:51

Abbreviation: mDIXON, modified Dixon.

Sagittal HT2‐FLAIR images following GBCA administration were acquired through intravenous administration of gadobutrol (Gadovist) at a standard dosage of 0.1 mmol/kg immediately postinjection (p.i.), as well as at 10 min p.i., and, in some cases, also at 3 h p.i. if the patient had agreed to an additional examination.

Thin‐sliced axial and coronal multiplanar reconstruction images were reconstructed using sagittal MR myelography and HT2‐FLAIR imaging datasets. Subtraction images were generated using pre‐ and postcontrast HT2‐FLAIR images.

### 2.3 Imaging Analysis

Two readers, one board‐certified radiologist with 8 years of neuroradiological experience and one board‐certified neuroradiologist with 10 years of neuroradiological experience, independently examined the noncontrast and contrast‐enhanced MR myelography images side by side to identify or disprove a CSF lamella. For each case, the following items were assessed: presence of an epidural fluid lamella on noncontrast MR myelography (yes/no/uncertain) and conspicuity of an epidural fluid lamella on ceHT2‐FLAIR compared to noncontrast MR myelography applying a conspicuity score (CS) (from −2 to +2; −2: missing epidural CSF lamella on ceHT2‐FLAIR compared to noncontrast MR myelography; −1: weaker presentation; 0: identical presentation; +1: higher conspicuity on ceHT2‐FLAIR; +2: conspicuous exclusively on ceHT2‐FLAIR). Finally, the reader rated whether intravenous contrast administration yielded an additional benefit compared to noncontrast MR myelography (no additional benefit; supporting information for decision on CSF leak presence; enabling diagnosis by information exclusively provided by ceHT2‐FLAIR).

### 2.4 Statistics

Statistical analyses were conducted using Microsoft Excel (Version 2007, Microsoft Corp., Redmond, USA) and SPSS statistical software (v27 and above, IBM Corp., Armonk, NY, USA, https://www.ibm.com/products/spss‐statistics). All applicable demographic and imaging data are given as mean ± standard deviation, unless otherwise specified.

## 3 Results

Patient characteristics are summarized in Table 1. All 10 patients presented with symptoms of IH, five after lumbar puncture and five spontaneously. All 10 patients underwent HT2‐FLAIR MRI with and without GBCA administration in addition to noncontrast MR myelography. Six of 10 patients received a blood patch after MRI ‐ two were targeted and four were nontargeted. Two cases did not receive a blood patch because the symptoms improved spontaneously.

The two readers rated in agreement when evaluating the CS and the diagnostic benefit of ceHT2‐FLAIR in all cases (for a summary of the results, see Table 3).

**TABLE 3:** Conspicuity score and diagnostic benefit of epidural fluid lamella assessment on ceHT2‐FLAIR MRI.

Patient#	Noncontrast MR myelography	ceHT2‐FLAIR		
	Epidural fluid lamella (yes/uncertain/no)	Conspicuity score (from −2 to +2)	Diagnostic benefit	Therapy
1	No	+2	Enabled diagnosis	No myeloCT performed, no blood patch performed
2	Yes	+1	Supporting information	Ventral predominance of CSF lamella → myeloCT performed → targeted blood patch performed
3	Yes	0	Confirmation	Ventral predominance of CSF lamella → myeloCT performed → targeted blood patch performed
4	Yes	0	Confirmation	Lateral predominance of CSF lamella → myeloCT performed → CT did not identify osteophyte → nontargeted blood patch performed
5	Yes	+1	Supporting information	No myeloCT performed → nontargeted blood patch performed
6	Yes	0	Confirmation	No myeloCT performed → nontargeted blood patch performed
7	Uncertain	+1	Enabled diagnosis	No myeloCT performed → nontargeted blood patch performed
8	No	0	Confirmation	No myeloCT performed → no blood patch performed
9	No	0	Confirmation	No myeloCT performed → no blood patch performed
10	Yes	0	Confirmation	No myeloCT performed → no blood patch performed

Abbreviations: ceHT2‐FLAIR, contrast‐enhanced heavily T2‐weighted fluid‐attenuated inversion recovery magnetic resonance imaging; CSF, cerebrospinal fluid.

### 3.1 CS

In one case, the presence of an epidural fluid lamella was not detected on noncontrast MR myelography but was detected on ceHT2‐FLAIR, resulting in a CS rating of 2. In three cases, the lamella depicted a higher conspicuity on ceHT2‐FLAIR (CS = 1). In six cases, the conspicuity was rated equal for both imaging methods (CS = 0), including two cases without a lamella on both noncontrast and contrast‐enhanced MR myelography. Thus, four out of eight cases (50%) with an epidural fluid lamella showed a higher conspicuity on ceHT2‐FLAIR images compared to noncontrast myelography.

### 3.2 Diagnostic Benefit of ceHT2‐FLAIR

**FIGURE 1:**
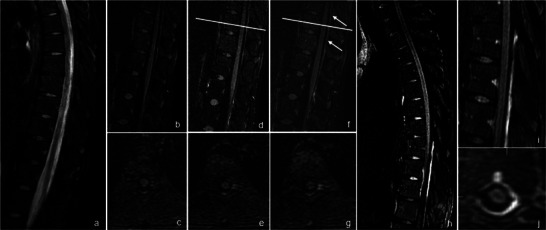
Presentation of a case with a cerebrospinal fluid (CSF) lamella exclusively identified on contrast‐enhanced heavily T2‐weighted fluid‐attenuated inversion recovery (ceHT2‐FLAIR) MRI, but not on noncontrast MR myelography. An 18‐year‐old female patient with intracranial hypotension and spinal muscular atrophy type 3 on intrathecal medication presented with continuous orthostatic headache following a recent difficult lumbar puncture. Sagittal noncontrast MR myelography including T2w modified DIXON (a) did not reveal any signs of a CSF leak. Therefore, the axial plane was not acquired. Note that flow artifacts in the thoracic spinal canal should not be misinterpreted as a CSF lamella. Sagittal and axial HT2‐FLAIR sequences are shown (b–j): noncontrast (b, c), immediately postinjection (d, e), 10 min postinjection (f, g), and 3 h postinjection (h–j). White arrows indicate an epidural fluid lamella with increasing contrast enhancement over time in the dorsal spinal canal (compare f and h). In particular, the ceHT2‐FLAIR at 3 h postinjection (h) shows a clearly visible epidural fluid collection within the spinal canal, predominantly in the dorsal lower thoracic and upper lumbar spine ([i] sagittal plane, zoomed; [j] confirmation in the axial plane).

**FIGURE 2:**
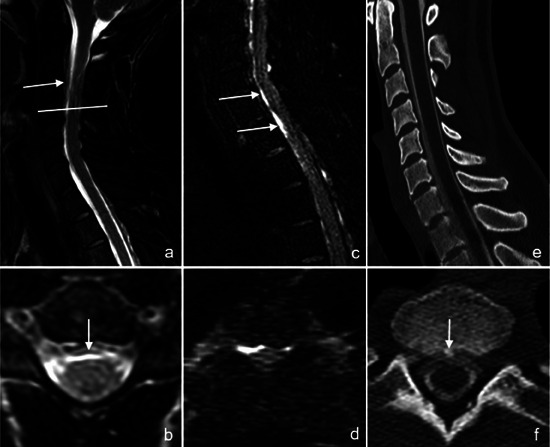
Presentation of a case in which the ventral predominance of a cerebrospinal fluid (CSF) lamella is better visualized on contrast‐enhanced heavily T2‐weighted fluid‐attenuated inversion recovery (ceHT2‐FLAIR) MRI than on noncontrast MR myelography. A 43‐year‐old female patient with intracranial hypotension (case #2) presented with therapy‐resistant, position‐dependent headache for 4 weeks without any history of trauma. On noncontrast MRI, the modified DIXON T2 sequence revealed a right lateral and ventral epidural fluid lamella in the sagittal plane (white arrow) (a), which was confirmed in the axial plane (white arrow) (b). On ceHT2‐FLAIR, the epidural CSF lamella is clearly visible despite motion artifacts and demonstrates strong contrast enhancement in both sagittal and axial planes immediately postinjection (white arrows) (c, d) with a ventral predominance. CT myelography in the sagittal and axial plane (e, f) confirmed the presence of the CSF lamella, and a causative osteophyte (white arrow) could be identified. The patient therefore received a targeted epidural blood patch. In this case, ceHT2‐FLAIR confirmed the findings of noncontrast MR myelography and visualized the predominance of a ventral CSF lamella even better.

**FIGURE 3:**
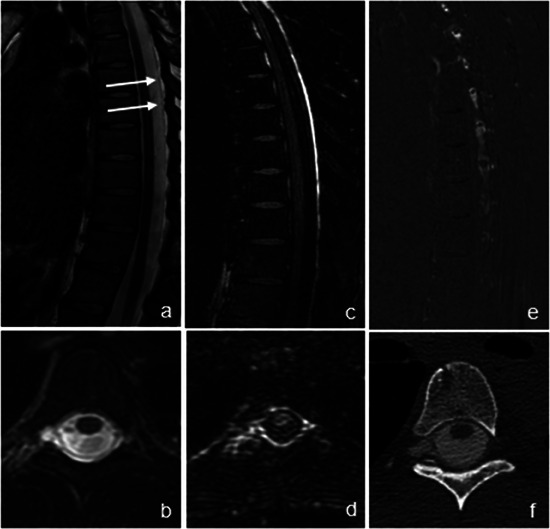
Presentation of a case in which the cerebrospinal fluid (CSF) lamella is clearly visualized on both intravenous contrast‐enhanced heavily T2‐weighted fluid‐attenuated inversion recovery (ceHT2‐FLAIR) MRI and noncontrast MR myelography. A 32‐year‐old female patient with intracranial hypotension (case #4) presented with therapy‐resistant, position‐dependent headache without any history of trauma. On noncontrast MRI, the modified DIXON T2 sequence revealed a right lateral and dorsal epidural fluid lamella in the sagittal plane (white arrows) (a), which was confirmed in the axial plane (b). On ceHT2‐FLAIR, the epidural CSF lamella is clearly visible despite motion artifacts and demonstrates strong contrast enhancement in both sagittal and axial planes immediately postinjection (c, d), again with right lateral predominance in the axial view (d) and the right paramedian subtraction image (e). CT myelography (f) confirmed the presence of the CSF lamella with right lateral predominance; however, a causative osteophyte could not be identified. The patient therefore received a nontargeted epidural blood patch. In this case, ceHT2‐FLAIR independently confirmed the findings of noncontrast MR myelography.

**FIGURE 4:**
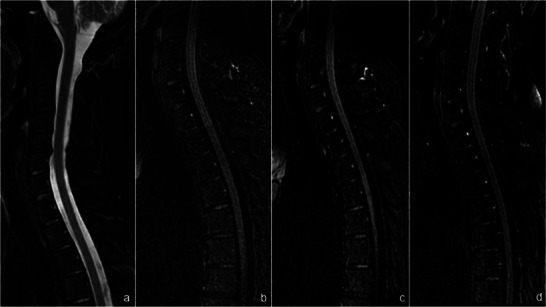
Presentation of a case without visible cerebrospinal fluid (CSF) lamella on either noncontrast MR myelography or contrast‐enhanced heavily T2‐weighted fluid‐attenuated inversion recovery (ceHT2‐FLAIR) MRI. A 47‐year‐old female patient with intracranial hypotension (case #8) presented with orthostatic headache and nausea upon standing, beginning 7 weeks after lumbar puncture. In the sagittal modified DIXON T2 sequence (a), no signs of a CSF leak were observed; therefore, no axial plane was acquired. Sagittal noncontrast HT2‐FLAIR (b) as well as postcontrast HT2‐FLAIR imaging at 10 min (c) and 3 h postinjection (d) confirmed the absence of a CSF lamella. CT myelography was deemed unnecessary, and the patient was treated with a nontargeted epidural blood patch.

In two out of 10 cases, both readers found that the ceHT2‐FLAIR sequence enabled diagnosis by providing information that was not available on noncontrast MR myelography. In one of these cases, the relevant finding was observed on the ceHT2‐FLAIR image acquired 3 h p.i. (illustrated in Figure 1). In another two cases (one of them illustrated in Figure 2), the readers found that ceHT2‐FLAIR provided supportive information. In all remaining cases (*n* = 6), they considered the inclusion of HT2‐FLAIR helpful, as it increased their confidence in determining the presence (Figure 3) or absence (Figure 4) of a CSF lamella.

Furthermore, beyond improved conspicuity, ceHT2‐FLAIR was found to offer diagnostic advantages, including detection of low‐flow CSF leaks not visible on noncontrast MR myelography, facilitation of optimal slice positioning for axial imaging, and assessment of the distribution pattern of CSF lamellae.

In summary, both readers found it beneficial to have the ceHT2‐FLAIR sequence available for the overall assessment in each of the 10 cases.

## 4 Discussion

This study investigated the benefit of intravenous GBCA‐enhanced MR myelography using ceHT2‐FLAIR compared to noncontrast MR myelography for detecting CSF lamellae in 10 individuals with IH.

The study found that in 50% of all cases with CSF lamellae, the lamellae showed greater conspicuity on ceHT2‐FLAIR compared to noncontrast MR myelography. Furthermore, both readers, with up to 10 years of neuroradiological experience, reported a diagnostic benefit from adding ceHT2‐FLAIR to the imaging workup in all 10 cases.

Therefore, ceHT2‐FLAIR could expand the diagnostic toolbox for CSF leak detection, especially since GBCAs might be administered to identify indirect signs of a CSF leak, such as diffuse dural enhancement, anyway. This approach avoids the need for radiation exposure, lumbar puncture, or off‐label intrathecal GBCA administration.

The benefits of intravenous contrast‐enhanced HT2‐FLAIR imaging compared to noncontrast MR myelography include increased conspicuity of CSF lamellae, identification of low‐flow CSF leaks potentially not visible on noncontrast MR myelography, facilitated determination of the optimal height for axial imaging, and assessment of the distribution pattern of CSF lamellae (e.g., ventral or dorsal predominance). Therewith, it helps to identify patients who would benefit from CT to detect degenerative causes of IH for targeted blood patching, while also providing an independent imaging modality to confirm the presence or absence of a CSF lamella.

Our study confirms the results of a previous investigation: Osawa et al. reported that ceHT2‐FLAIR imaging was superior in the visualization of CSF lamellae compared to MR myelography [21]. The reason for the superiority over MR myelography could be that unsuppressed epidural fat exhibits hyperintensity on MR myelography exactly as CSF, which can be misinterpreted as CSF lamellae. Furthermore, linear CSF flow artifacts might mimic epidural fluid lamellae.

Noncontrast MR myelography predominantly depends on heavily T2‐weighted sequences that amplify CSF signal while dampening the surrounding background signal. This method has gained growing attraction in clinical environments, utilizing both 2D and 3D sequences [12, 13, 22–25]. However, a CSF lamella might be difficult to distinguish from intradural CSF. In contrast, the advantage of employing a 3D HT2‐FLAIR sequence is the enhanced contrast specifically between CSF lamellae and the surrounding anatomical structures, including intradural CSF, while it furthermore diminishes artifacts linked to CSF movement. Due to the high spatial resolution and the 3D approach, image data can be reconstructed, offering visual flexibility.

Another competing imaging technique is intrathecal GBCA‐enhanced MR myelography. However, it carries the, albeit very small, risk of neurotoxic side effects from intrathecal GBCA use, as well as risks related to the lumbar puncture. It is therefore no longer widely used in this context, given its relatively low yield in detecting CSF leaks [26, 27].

We deem ceHT2‐FLAIR imaging a truly complementary approach compared to noncontrast MR myelography, as noncontrast imaging anatomically visualizes the fluid lamella, while ceHT2‐FLAIR adds a functional component, namely, the extravasation of GBCA in the area of the lamella. The actual CSF leak—the exact point of CSF egress into the epidural or paraspinal space—could not be identified on ceHT2 FLAIR, as the entire lamella in most cases enhanced immediately after contrast injection.

While the actual pathophysiology for GBCA enhancement in the area of the lamellae is not known yet, our hypothesis is that GBCA extravasation occurs in the area of the dural tear, as the dura is highly vascularized. Considering that in IH patients the CSF predominantly flows from the subarachnoid space to the extra‐arachnoid space via dural tears, it is conceivable that extravasated contrast agent might preferentially accumulate in the extra‐arachnoid area, whereas extravasated GBCA in the subarachnoid compartment is diluted by motion and volume of subarachnoid CSF [28].

In cases with clear conspicuity of a CSF lamella on noncontrast MR myelography, intravenous GBCA administration is unnecessary. However, it should be noted that ceHT2‐FLAIR might be applied to identify low‐flow leaks, especially if GBCAs are administered for other reasons.

Our study presents certain constraints: The small sample size is a limitation. Furthermore, ceHT2‐FLAIR imaging provides fewer anatomical details compared to fat‐suppressed T2‐weighted imaging, primarily because it suppresses the background signal, except for the contrast material.

Ultimately, the HT2‐FLAIR sequence was acquired with a slightly different resolution compared to noncontrast myelography (lower in‐plane resolution but smaller slice thickness). However, the deviation is minor and unlikely to have had a meaningful impact on the results.

In conclusion, this study highlights the value of intravenous contrast‐enhanced MR myelography for detecting CSF lamellae in patients with IH.

Acquisition of a ceHT2‐FLAIR sequence can be considered a valuable addition to the diagnostic algorithm for IH—particularly in patients already receiving GBCAs for the evaluation of indirect signs of IH, in those suspected of having low‐flow leaks, or in cases where iodinated contrast media are contraindicated.

By enhancing the visualization of CSF lamellae while reducing the need for further invasive diagnostic procedures, this imaging modality is safe and complementary to noncontrast MR myelography in the diagnostic workup of IH.

## Disclosure

The authors disclose commercial considerations in the past 2 years, such as equity interests, patent rights, or corporate affiliations, including director roles, stock ownership, consultantships, speaking fees, and research support, for any product or process relevant to the submission.

## Conflicts of Interest

K.D., D.P., A.R., and R.H. are co‐founder of relios.vision GmbH.
